# And the Oscar Goes to Peripheral Blood Film for the Detection of Lead Poisoning in a Complicated Toxic Patient: A Case Report with a Review of Laboratory Clues

**DOI:** 10.1155/2022/9238544

**Published:** 2022-02-23

**Authors:** Amir Zamani, Ehsan Sarraf Kazerooni, S. Saeed Kasaee, Mohammad Hossein Anbardar, Sahand Mohammadzadeh, Golsa Shekarkhar, Neda Soleimani

**Affiliations:** ^1^Department of Pathology, Shiraz Transplant Center, Abu Ali Sina Hospital, Shiraz University of Medical Sciences, Shiraz, Iran; ^2^Diagnostic Laboratory Sciences and Technology Research Center, School of Paramedical Sciences, Shiraz University of Medical Sciences, Shiraz, Iran; ^3^Department of Pathology, Shiraz Medical School, Shiraz University of Medical Sciences, Shiraz, Iran; ^4^Pathology Department, Molecular Pathology, Raz Pathobiology Lab, Shiraz, Iran

## Abstract

**Background:**

Peripheral blood smear examination is an invaluable laboratory test, which provides the complete hematologic and/or nonhematologic picture of a case. In addition to verifying the results of automated cell counters, it has the potential to identify some pathologic and morphologic changes that remain hidden using the cell counters alone. *Case Presentation*. A 40-year-old man with a three-year history of alcohol intake and marijuana abuse presented with severe lower extremities of the bone and abdominal pain. Physical examination showed high blood pressure, high pulse rate, and abdominal tenderness. He underwent extensive laboratory and imaging tests, and cholecystectomy and bone marrow studies were associated with no definite diagnosis. Right after all these invasive, expensive, and time-consuming investigations during a month, finding coarse basophilic stippling in the red blood cells in the peripheral blood smear by an expert led to the final diagnosis. Elevated blood lead level and the presence of ring sideroblasts in the bone marrow study confirmed the diagnosis of lead poisoning, and the patient responded well to chelator therapy in a short period.

**Conclusion:**

This case clearly showed the value of peripheral blood smear review and its impact on patient care. In order not to lose the cases, laboratories are recommended to design their own policy for peripheral blood smear review. The peripheral blood smear is the fastest, simplest, and most available screening test, which can prevent many misdiagnoses and malpractices. It provides rich morphological information, among which basophilic stippling is highly suggestive of lead poisoning.

## 1. Background

A manual blood smear review is defined as the thorough and careful microscopic analysis of a well-prepared and stained smear of peripheral blood, with the objective of seeking morphological changes relevant to the diagnosis and monitoring of patients [1]. Laboratory-initiated examinations of blood smears for patients with anemia are usually the result of a laboratory policy, according to which a blood smear is ordered whenever the hemoglobin concentration is unexpectedly low. In these cases, despite the wealth of the captured information from modern automated cell counters, including red cell count, mean cell volume (MCV), mean cell hemoglobin (MCH), mean cell hemoglobin concentration (MCHC), and red cell distribution width (RDW), there are still morphologic abnormalities that are critical in the differential diagnosis of anemia and can be determined only from a blood smear. The detection of variations in the cell shape and red cell inclusions, such as Howell–Jolly bodies, Pappenheimer bodies, and basophilic stippling or punctate basophilia, is highly important. The latter can be suggestive of lead poisoning according to the clinical data [2].

## 2. Case Report

A 40-year-old man was transferred to our center at Abu Ali Sina Hospital, Shiraz, Iran, to be evaluated for severe bone pain and abdominal pain for a month, which was worsened one week ago. It was accompanied by nausea, vomiting, and constipation. The patient was also complaining of severe weight loss (15 kg during a month). His past medical history was not significant, and he was not taking any medication. He was a fast-food worker with a history of excessive alcohol intake over three years, with no periods of abstinence. He was drinking 1-2 liters of homemade alcohol (using a traditional container for distillation) daily until few days prior to admission. Additionally, he reported a history of on and off synthetic marijuana abuse until three days prior to admission. The bone pain was mostly limited to the lower extremities, and the abdominal pain was severe with a colicky pattern and radiation to both scrotums. Physical examination was significant for high blood pressure (180/100 mmHg), tachycardia (108/min), low-grade fever (37.9°C), and pallor with abdominal and scrotal tenderness. There was no gingival hyperpigmentation, and all deep tendon reflexes were unremarkable.

**Figure 1:**
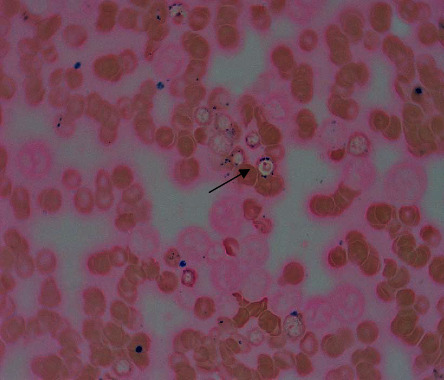
Bone marrow smear showing that about 10% of erythroid precursors are ring sideroblasts (arrow) (Perl's Prussian blue stain, ×1000).

**Figure 2:**
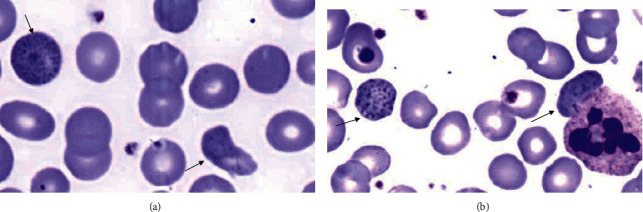
(a, b) Peripheral blood smear with basophilic stippling of red blood cells (arrows) (Wright–Giemsa, ×1000).

Laboratory investigations at presentation showed moderate microcytic and hemolytic anemia in addition to significantly elevated liver enzymes, including aspartate aminotransferase (AST) and alanine aminotransferase (ALT). Abdominopelvic sonography and CT scan showed biliary sludge and small liver hemangioma, respectively. Other laboratory and imaging investigations (color Doppler sonography of the mesenteric artery and vein, upper gastrointestinal endoscopy, and colonoscopy) were unremarkable. He underwent cholecystectomy with the impression of acute cholecystitis, and the microscopic pathologic diagnosis was just chronic cholecystitis without cholelithiasis. Due to the presence of unexplained anemia and severe bone pain, a bone marrow study was done for him to rule out leukemia, which was in favor of myelodysplastic syndrome (MDS) with 10% ring sideroblasts (Figure 1). The cytogenetic study was normal. Complete blood count (CBC) test with peripheral blood smear (PBS) checking (according to our policy in the hematology laboratory) was done for the patient several times at presentation and during hospitalization. Peripheral blood film examinations, stained by the Wright–Giemsa method, revealed mild to moderate anemia, microcytic hypochromic red blood cells with anisocytosis, polychromasia, and also frequent coarse basophilic stippling of the red cells (Figures 2(a) and 2(b)); the last one was missed at the first PBS review. Regarding the presence of ring sideroblasts in the bone marrow report, the possibility of lead poisoning was considered, and surprisingly, the blood lead level (measured on K2EDTA-whole blood by atomic absorption spectrophotometry) was markedly raised (151 *µ*g/dL; reference range was 10 *µ*g/dL). Since there was no occupational exposure, the traditional distillation dish (made of copper and lead) was traced as the possible source of lead.

The patient stopped drinking alcohol and marijuana abuse. Chelating therapy with succimer was initiated with a dose of 500 mg (5 capsules) daily, and after four weeks, the blood lead level declined to 25 *µ*g/dl, and he had a weight gain of 6 kg. All signs and symptoms were removed, and all laboratory findings changed to normal condition. Additionally, no clinical or laboratory deficits were noted during the 6-month follow-up visits. Informed consent was requested and obtained from the patient for publishing the case report and the accompanying images. Although some reports are supporting the correlation between hypertension and lead poisoning, in our case, marijuana withdrawal could also be the cause of sympathetic overactivity [3].  Table 1 shows the transition of laboratory data from the first presentation to recovery time.

## 3. Discussion

CBC is the most commonly ordered test in hospitalized patients. Modern automated blood cell counters produce a detailed report on the red cells, white cells, and platelets, with differential white blood cell counts. A drop of the remaining blood could then be smeared and stained [4]. Microscopic examination of this smear under the expert eye yields useful information and further details regarding all formed elements of blood [5]. “More information can be gained from examining the blood smear than any other single hematologic procedure,” Jandl in 1987 said [6]. This shows the value of PBS review in medical management. Initiation of a PBS review depends on the laboratory policy. It can be a clinical request by the attending clinician due to clinical suspicion or can be initiated by the laboratory due to clinical information, numerical deviations in the automated counts, flags raised by the analyzers, or based on a designed protocol in the laboratory (based on the workload of the laboratory) [4, 7]. For certain disorders, for instance, hematologic malignancies or parasitic infestations, examination of the PBS can be diagnostic. For others, such as our case, the PBS provides important clues in the clinical management and determines which diagnostic tests are indicated [2].

Among all useful data obtained from the blood smear, valuable information about the red blood cell size, shape, staining (color), and intracellular inclusions can be obtained. Red cell inclusions often result from defective maturation of the erythrocytes, oxidant injury to the cells, or infections and are almost always indicative of some sort of pathology [7].

Basophilic stippling (punctuate basophilia) is well-known red cell inclusions. The occurrence of basophilic stippling is caused by aggregates of ribosomes or fragments of ribosomal RNA precipitating throughout the cytoplasm of erythrocytes. These inclusions stain a deep blue color with Wright's stain, and unlike other erythrocyte inclusions, they lack polarity and are dispersed throughout the cell. The inclusions might have a coarse or fine appearance, and these different morphologies can be linked to particular disease processes [8, 9].

Fine basophilic stippling may be seen artifactually and is linked to increased production of the red cells, while coarse basophilic stippling is always clinically significant. Classically, coarse basophilic stippling is associated with heavy metal toxicity with a focus on lead poisoning. Patients with anemia secondary to hemoglobinopathies such as thalassemia and sickle cell disease can demonstrate basophilic stippling that is finer than that seen in lead toxicity. Lead can increase membrane permeability; furthermore, it can alter erythrocyte morphology, giving rise to macrocytic and echinocytic forms as well as basophilic stippling. Lead toxicity causes coarse basophilic stippling due to suppression of pyrimidine 5′-nucleotidase, which prevents ribosomal RNA breakdown in circulating erythrocytes. In lead poisoning, basophilic stippling is a pathognomonic feature [7–11].

Lead exposure usually occurs through contaminated air and food. Excess lead affects almost every organ in the body. The major anatomic targets are the blood (bone marrow), nervous system, gastrointestinal tract, and kidneys. Lead has a high affinity for sulfhydryl groups and interferes with the enzymes involved in heme synthesis. Iron incorporation into heme is impaired, leading to microcytic hypochromic anemia with distinctive punctate basophilic stippling of the red cells. Lead also inhibits sodium- and potassium-dependent ATPases in the cell membranes, which may increase the fragility of red cells, causing hemolytic anemia [12].

The classic form of lead neuropathy consists of weakness that primarily involves the wrist and finger extensor (wrist drop and foot drop). Excess lead could induce “lead lines” formation in epiphyses of the bone and gum. The gastrointestinal tract is also a locus for major clinical manifestations. Lead “colic” is characterized by extremely severe, poorly localized abdominal pain, and finally, because the excretion of lead occurs through the kidneys, exposures may cause damage to the proximal tubules, interstitial fibrosis, and possibly renal failure [12].

Since lead poisoning is a condition with potential multiorgan damage, it can be traced in the human body with various laboratory tests. The half-life of lead in the blood and bone is 35 days and 32 years, respectively; thus, the blood lead level just reflects the current and recent exposure [5, 13].  Table 2 shows different laboratory tests, their properties, and changes in cases of lead poisoning. Elevated blood lead (>10 *µ*/dL) and red cell-free protoporphyrin levels (>50 *µ*g/dL) or, alternatively, zinc protoporphyrin levels, are required for definitive diagnosis [5].

Impairment of the hematological system is one of the earliest signs of lead accumulation and could be characteristic. Even in milder cases of lead exposure, anemia may be the only obvious abnormality [5]. The PBS is the fastest, simplest, and cost-effective test, which can also be made and examined at emergency and screening rooms at hospitals and could prevent unnecessary laboratory tests and other invasive diagnostic procedures, including inappropriate laparotomy [17]. Because this process is among the most time-consuming ones in hematology laboratories and requires high technical competence to minimize the errors of subjectivity, each laboratory should make its policy to review the blood smears. Providing clinical data including occupational and environmental health history through communication between the clinicians and the laboratory team could lead to a more rapid and accurate diagnosis [18].

Our patient was a case of chronic lead poisoning with atypical symptoms and signs, such as severe bone pain and highly elevated liver enzymes, which caused delayed diagnosis. In this case, a lot of laboratory and imaging workups, including hematologic tests (and also the initial PBS) failed to reach a correct diagnosis, while the second PBS review by an expert clarified the long and painful story. We believe that even the first PBS review may discover the problem if it is reviewed accurately and/or is provided with typical clinical data.

## 4. Conclusion

This case emphasizes that all patients with unknown diseases should be evaluated in terms of PBS at the first workup, and the PBS in all patients (hematologic and nonhematologic) should be reviewed systematically regarding all blood elements. Lead poisoning is more common than our estimation. As clinical manifestations are nonspecific and may be misdiagnosed with other pathophysiological conditions, at least it should be included in differential diagnosis lists of adult unexplained anemia (especially in the setting of matched clinical data), and basic initiative laboratory tests, such as PBS, could rule out the need for invasive, expensive, and time-consuming procedures.

## Figures and Tables

**Table 1:** The initial and posttreatment results (after 4 weeks) of the laboratory tests.

Parameter	Initial result	Posttreatment results	Reference range (unit)
AST	130	18	3–40 (IU/L)
ALT	608	24	3–40 (IU/L)
ALP	730	121	80–306 (IU/L)
Total bilirubin	0.95	0.46	0.2–1 (mg/dL)
Direct bilirubin	0.52	0.14	0.1–0.3 (mg/dL)
ESR	10	8	0–20 (mm/Hr)
WBC	13.8	9.09	4.5–11 (×10^3^/*µ*L)
HB	10.1	14.8	14–18 (g/dL)
MCV	76.04	82.8	80–96 (fl)
MCH	24.69	27.2	27–33 (pgr)
MCHC	32.48	32.8	32–36 (g/dL)
RDW-CV	16.9	14.6	11–13 (%)
PLT	573	357	150–450 (×10^3^/*µ*L
Reticulocyte count	2	1	0.5–1.5 (%)
Basophilic stippling	++	Absent	—

Blood sugar, blood urea nitrogen (BUN), creatinine, sodium, potassium, calcium, magnesium, phosphorus, rheumatologic tests, HBS antigen, anti-HCV antibody, anti-HAV antibody (IgM), anti-HIV antibody, and urinalysis were negative or within the normal ranges all the time. AST: aspartate aminotransferase; ALT: alanine aminotransferase; ALP: alkaline phosphatase; ESR: erythrocyte sedimentation rate; WBC: white blood cell; HB: hemoglobin; MCV: mean corpuscular volume; MCH: mean corpuscular hemoglobin; MCHC: mean corpuscular hemoglobin concentration; RDW-CV: red cell distribution width-coefficient of variation; PLT: platelet.

**Table 2:** Change of different laboratory tests in lead poisoning.

Test	Specimen	Diagnostic clue	Mechanism
HB	Whole blood	Decreased	Microcytic and hemolytic anemia [5]
MCV	Whole blood	Decreased	Iron incorporation into heme is impaired [5]
MCH	Whole blood	Decreased	Iron incorporation into heme is impaired [5]
PBS	Whole blood	Coarse basophilic stippling—Cabot rings	Instability of RNA and remaining microtubules, respectively, due to abnormal erythropoiesis [5]
Reticulocyte count	Whole blood	Increased	Hemolytic anemia due to the inhibition of Na-K-dependent ATPases in cell membranes and increased fragility [12]
BM iron stain	BM aspiration smear	Ring sideroblasts	Iron accumulation in the mitochondria [12]
Blood lead level	Whole blood	Increased	*∗*
Zinc protoporphyrin	Whole blood	Increased	Zinc protoporphyrin is formed instead of heme [12]
Free red cell protoporphyrin	Whole blood	Increased	It is a product of zinc protoporphyrin [12]
LDH	Serum/plasma	Increased	Hemolytic anemia [14]
AST/ALT	Serum/plasma	Increased	Increasing oxidative stress [15]
BUN/Cr	Serum/plasma	Increased	Proximal tubular dysfunction and Fanconi-type syndrome [16]
Urine glucose	Urine	Increased (positive)	Proximal tubular dysfunction and Fanconi-type syndrome [16]

^*∗*^ indicates not applicable. HB: hemoglobin; MCV: mean corpuscular volume; MCH: mean corpuscular hemoglobin; PBS: peripheral blood smear; BM: bone marrow; LDH: lactate dehydrogenase; AST: aspartate aminotransferase; ALT: alanine aminotransferase; BUN: blood urea nitrogen; Cr: creatinine.

## Data Availability

All data generated or analysed during this study are included within this published article.

## Abbreviations

ALT: Alanine aminotransferase
ALP: Alkaline phosphatase
AST: Aspartate aminotransferase
BUN: Blood urea nitrogen
BM: Bone marrow
CBC: Complete blood count
Cr: Creatinine
ESR: Erythrocyte sedimentation rate
HB: Hemoglobin
LDH: Lactate dehydrogenase
MCH: Mean cell hemoglobin
MCHC: Mean cell hemoglobin concentration
MCV: Mean cell volume
MDS: Myelodysplastic syndrome
PBS: Peripheral blood smear
PLT: Platelet
RDW: Red cell distribution width
WBC: White blood cell.
